# Enhancing foveal avascular zone analysis for Alzheimer’s diagnosis with AI segmentation and machine learning using multiple radiomic features

**DOI:** 10.1038/s41598-024-51612-8

**Published:** 2024-01-22

**Authors:** Je Moon Yoon, Chae Yeon Lim, Hoon Noh, Seung Wan Nam, Sung Yeon Jun, Min Ji Kim, Mi Yeon Song, Hyemin Jang, Hee Jin Kim, Sang Won Seo, Duk L. Na, Myung Jin Chung, Don-Il Ham, Kyungsu Kim

**Affiliations:** 1 Department of Ophthalmology, Samsung Medical Center, Sungkyunkwan University School of Medicine, Seoul, 06351 Republic of Korea; 2 Department of Medical Device Management and Research, SAIHST, Sungkyunkwan University, Seoul, 06351 Republic of Korea; 3 Hangil Eye Hospital, 35 Bupyeong-daero, Bupyeong-gu, Incheon, 21388 Republic of Korea; 4 Department of Ophthalmology, Catholic Kwandong University College of Medicine, 35 Bupyeong-daero, Bupyeong-gu, Incheon, 21388 Republic of Korea; 5 Alzheimer’s Disease Convergence Research Center, Samsung Medical Center, Seoul, Republic of Korea; 6 Department of Neurology, Samsung Medical Center, Sungkyunkwan University School of Medicine, Seoul, 06351 Republic of Korea; 7 Neuroscience Center, Samsung Medical Center, Seoul, Republic of Korea; 8 Department of Digital Health, Samsung Advanced Institute for Health Sciences & Technology, Sungkyunkwan University, Seoul, Republic of Korea; 9 Happymind Clinic, Seoul, Republic of Korea; 10 Medical AI Research Center, Research Institute for Future Medicine, Samsung Medical Center, Seoul, 06351 Republic of Korea; 11 Department of Data Convergence and Future Medicine, Sungkyunkwan University School of Medicine, Suwon, 16419 Republic of Korea; 12 Department of Radiology and AI Research Center, Samsung Medical Center, Sungkyunkwan University School of Medicine, Seoul, 06351 Republic of Korea

**Keywords:** Dementia, Neuroscience, Biomarkers, Mathematics and computing, Machine learning, Biophotonics, Optical techniques

## Abstract

We propose a hybrid technique that employs artificial intelligence (AI)-based segmentation and machine learning classification using multiple features extracted from the foveal avascular zone (FAZ)—a retinal biomarker for Alzheimer’s disease—to improve the disease diagnostic performance. Imaging data of optical coherence tomography angiography from 37 patients with Alzheimer’s disease and 48 healthy controls were investigated. The presence or absence of brain amyloids was confirmed using amyloid positron emission tomography. In the superficial capillary plexus of the angiography scans, the FAZ was automatically segmented using an AI method to extract multiple biomarkers (area, solidity, compactness, roundness, and eccentricity), which were paired with clinical data (age and sex) as common correction variables. We used a light-gradient boosting machine (a light-gradient boosting machine is a machine learning algorithm based on trees utilizing gradient boosting) to diagnose Alzheimer’s disease by integrating the corresponding multiple radiomic biomarkers. Fivefold cross-validation was applied for analysis, and the diagnostic performance for Alzheimer’s disease was determined by the area under the curve. The proposed hybrid technique achieved an area under the curve of $72.2\pm 4.2$%, outperforming the existing single-feature (area) criteria by over 13%. Furthermore, in the holdout test set, the proposed technique exhibited a 14% improvement compared to single features, achieving an area under the curve of 72.0± 4.8%. Based on these facts, we have demonstrated the effectiveness of our technology in achieving significant performance improvements in FAZ-based Alzheimer’s diagnosis research through the use of multiple radiomic biomarkers (area, solidity, compactness, roundness, and eccentricity).

## Introduction

Alzheimer’s disease (AD) is the most common type of dementia, affecting an estimated 5.4 million people in the United States^1^. With an estimated 13.8 million people affected by AD by 2050, health and societal expenses are predicted to notably increase^2^. The identification of biomarkers for early diagnosis and recruitment in interventional clinical trials has become a priority given the increasing prevalence of AD and lack of efficient therapeutic options. Current AD diagnosis is constrained by its expensive equipment (e.g., magnetic resonance imaging, positron emission tomography [PET]), invasiveness (e.g., extraction of cerebrospinal fluid [CSF]), insufficient specificity and sensitivity (e.g., genetic markers, serum amyloid), length of examination, accessibility to specialists, and neuropsychological testing^3^. For effective risk screening, the demand for faster, more accessible, and less invasive diagnostic tests is largely unmet.

AD is characterized by neuronal death, brain atrophy, extracellular deposition of beta-amyloid plaques, and intracellular accumulation of neurofibrillary tangles^4,5^. In addition, changes to the cerebral vasculature, such as cerebral amyloid angiopathy, atherosclerosis, and arteriosclerosis, reduced capillary density, and changed capillary morphology, have also been observed^6–9^. However, current in vivo imaging modalities fail to detect cerebral microvascular alterations.

The retina is considered an extension of the central nervous system, because the retina originates as outgrowth of the developing brain^10^. Hence, the retina may be a mirror of the brain. As sources of biomarkers for AD, retinal imaging modalities such as optical coherence tomography and fundus photography have been the subject of systematic reviews and meta-analyses^11,12^. Furthermore, unlike in vivo imaging of cerebral microvasculature, retinal microvasculature can be detected by optical coherence tomography angiography (OCTA), which provides high-resolution images of the retinal microvasculature and choroid^13^.

The foveal avascular zone (FAZ) is a region surrounding the fovea and devoid of retinal capillaries, and it can be imaged using OCTA. Although a meta-analysis has revealed an increase in the FAZ area in AD^14^, heterogeneity and conflicting results have been observed among studies. Recent research has shown that the FAZ shape in OCTA images is a reliable indicator of retinal disorders. For instance, the FAZ circularity and axial ratio are considerably different in eyes with diabetic retinopathy and normal eyes^15,16^, and the FAZ circularity is significantly lower in eyes with glaucoma presenting central visual field deficits than in eyes with peripheral visual field defects^17^. However, previous studies on AD using OCTA have mainly focused on the FAZ area, but its shape has mostly been neglected^14^.

In this study, we analyzed multiple FAZ features using radiomics-based machine learning (ML) for AD diagnosis. In addition, we investigated the diagnostic ability of other features when combined with the FAZ area. Finally, we developed a diagnosis method for AD that combines artificial-intelligence (AI)-based FAZ segmentation and an ML model for processing multiple radiomic features.

Our contributions are summarized as follows:

- The validity of existing representative techniques for AD diagnosis considering the FAZ area is verified with data from Korean patients collected at our hospital, showing an area under the curve (AUC) of 60%.
- Existing techniques do not use features other than the FAZ area, but *our technique includes other key FAZ features*, thus improving the AD diagnostic performance by more than 10% in AUC up to 72%.
- Unlike existing techniques that require manual annotations from specialists to extract the FAZ region, we apply *automatic AI-based segmentation that can promote the diagnostic performance*. Hence, a fully automatic technique for AD diagnosis from OCTA scans is obtained.
- We demonstrate that the proposed technique outperforms representative AI models for the differentiation of AD using OCTA scans as input. This demonstrates the usefulness of our *hybrid* diagnosis technique that combines AI-based segmentation and ML-based classification for Alzheimer’s using multiple radiomic features.

## Materials and methods

### Ethical approval

All authors of this study confirm that all methods or experiments were performed in accordance with the Declaration of Helsinki and the relevant guidelines and regulations provided by the policies of the Nature Portfolio journals. This study was approved by the Institutional Review Board of the Samsung Medical Center (IRB number: SMC 2021-05-073). Written informed consent from the patients was waived by the Institutional Review Board (Samsung Medical Center, Seoul, Republic of Korea) because we used anonymized retrospective data.

### Study participants

All participants underwent amyloid PET and brain MRI at the memory clinic in the Department of Neurology at SMC in Seoul, South Korea^18^. As previously described^19^, all participants underwent comprehensive dementia evaluation, including a standardized neuropsychological test (Seoul Neuropsychological Screening Battery, 2nd edition^20^), blood tests including APOE genotyping, and brain MRI. We excluded participants who had any of the following conditions: (1) white matter hyperintensities due to etiologies other than vascular pathology, including radiation injury, multiple sclerosis, leukodystrophy, or metabolic/toxic disorders; (2) traumatic brain injury; (3) normal pressure hydrocephalus; (4) territorial infarction; (5) neurodegenerative disorders other than AD or ischemic etiologies such as progressive supranuclear palsy, corticobasal syndrome, frontotemporal dementia, or Lewy body/Parkinson disease dementias; or (6) rapidly progressive dementias and treatable dementias. All participants with normal controls (NCs) fulfilled the following criteria: (1) subjective memory complaints by participants or caregivers; (2) no objective cognitive dysfunction, as assessed by scores from evaluations on any cognitive domain; (3) no history of medical diseases likely to affect cognitive function; and (4) no significant impairment in activities of daily living. All patients diagnosed with MCI fulfilled Petersen criteria for MCI^21^. Patients with dementia satisfied diagnostic criteria for dementia according to the DSM-IV^22^.

### Optical coherence tomography angiography acquisition and clinical data

**Figure 1:**
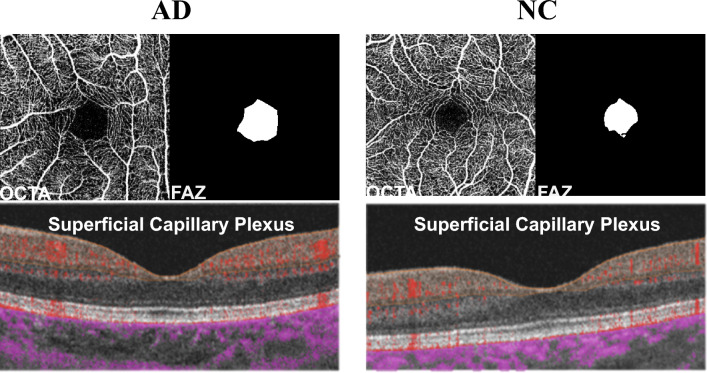
OCTA scans in superficial capillary plexus layer for AD and NC. We aim to determine whether various FAZ features extracted from these scans contribute to AD diagnosis.

All participants underwent OCTA scans^25^ of the superficial capillary plexus layer^26^ at Samsung Medical Center, Gangnam-Gu, Seoul, South Korea, between November 2021 and February 2023. OCTA was performed by an expert technician. The OCTA scanning protocol used was a 3 $\times$ 3 mm$^2$ volume scan centered on the fovea (DRI OCT Triton, Topcon, Japan). During data collection, we acquired clinical information of patients including age, sex, presence of hypertension or diabetes, education level, mini-mental state examination score^23^, and clinical dementia rating^24^, as specified in Table 1. From the clinical information, we used age and sex, which can affect the FAZ shape^27^ and measured without any additional cost, along with OCTA scans as inputs for the proposed diagnosis technique. We obtained a total of 170 OCTA scan sets from 85 participants, as shown in Fig. 1. In addition, the 170 OCTA scans are divided into the training set and the holdout test set. From the scan sets, 25 scans were excluded from the training set, and 15 were excluded from the holdout test set. The exclusion criterion was considerable noise or low image quality, depending on the scanning environment^28^. Hence, the OCTA training set includes a total of 85 scans, with 31 scans from AD cases and 54 scans from NC cases, while the holdout test set consists of 45 scans, with 29 scans from AD cases and 16 scans from NC cases. In addition, we aimed to maximize the separation between the training dataset and the holdout dataset by collecting them at different times, although both were from a single institution.

**Table 1:** Characteristics of data sets.

Data type	Patient-wise training set (*n* = 55)			Patient-wise holdout test set (*n* = 30)		
Data collection period	November 2021–September 2022			July 2022–February 2023		
Disease type	AD (*n* = 20)	NC (*n* = 35)	*P*-value	AD (*n* = 17)	NC (*n* = 13)	*P*-value
Age (years)	$65.7\pm 7.9$	$69.8\pm 7.1$	0.186	$66.8\pm 4.9$	$73.7\pm 5.5$	$<0.005$
Sex (Male, %)	$45\%$ (*n* = 9)	$28.5\%$ (*n* = 10)	0.185	$58.8\%$ (*n* = 10)	$46.1\%$ (*n* = 6)	0.164
Mini-mental state examination score^23^	$18.4\pm 5.5$	$28.7\pm 1.6$	$< 0.005$	$20.1\pm 1.3$	$28.0\pm 2.4$	$<0.005$
Study period (months)	$12.0\pm 3.7$	$12.0\pm 5.0$	0.996	$13.5\pm 4.7$	$11.0\pm 4.9$	0.107
Clinical dementia rating^24^	$0.9\pm 0.9$	$0.1\pm 0.3$	$< 0.005$	$0.6\pm 0.2$	0.5	$<0.005$
Patients w/hypertension (%)	$30\%$ (*n* = 6)	$37.1\%$ (*n* = 13)	0.789	$29.4\%$ (*n* = 5)	$35.7\%$ (*n* = 5)	0.218
Patients w/diabetes (%)	$15\%$ (*n* = 3)	$20\%$ (*n* = 7)	0.787	$29.4\%$ (*n* = 5)	$7.1\%$ (*n* = 1)	0.139

### Dataset split

The dataset was divided into training and holdout test sets for each image before model selection. The training set was divided on a per-image basis for the fivefold cross-validation experiment, and the holdout test set was used exclusively for holdout testing without overlap with the training set. A summary of the data splitting used in the fivefold cross-validation and the holdout test is shown in Table 2.

**Table 2:** Summary of fivefold cross-validation and holdout test the data distribution of FAZ binary mask images.

Method	Fivefold cross-validation		Holdout test
Data type	Training set (*n* = 85)		Holdout test set (*n* = 45)
Split	Train	Test	Test
Fold 1	70	15	45 (64%)
Fold 2	68	17	
Fold 3	69	16	
Fold 4	67	18	
Fold 5	66	19	

### Existing and proposed techniques for AD diagnosis

**Figure 2:**
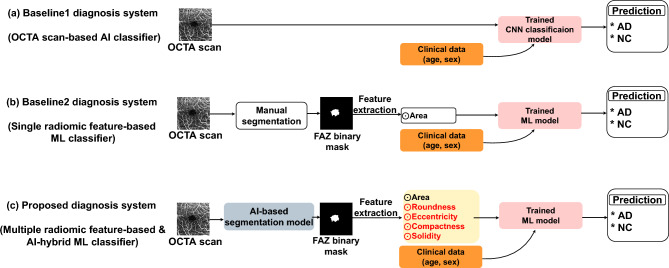
Comparison of AD diagnosis techniques using FAZ analysis on OCTA scans. (a) Baseline 1—AI classifier with OCTA input, (b) baseline 2—ML classifier using single radiomic FAZ feature with manual FAZ segmentation, and (c) proposed technique—ML classifier with AI-based automatic FAZ segmentation using multiple radiomic FAZ features.

In this section, we describe the inference of current AD diagnosis techniques by FAZ analysis using OCTA scans and compare these techniques with our proposal, as shown in Fig. 2. Existing AD diagnosis techniques using FAZ features include deep learning classification using OCTA scans (baseline 1) and radiomics based on a single FAZ feature (baseline 2).

#### Baseline 1: OCTA AI-based classifier

As shown in Fig. 2a, baseline 1^29^ receives an OCTA scan as input for AI-based classification and learns to diagnose AD by binary classification between AD and NC. We use convolutional neural networks (CNNs)^30^, which are the gold standard models, for classification and design of a multimodal AI network that uses clinical and image information by expanding the input vector to receive two additional clinical datapoints in a fully connected network within the target CNN.

Baseline 1 learns to classify AD and NC through a CNN classifier using OCTA scan *O* and two clinical datapoints as follows:

1 $$\begin{aligned} Baseline_{1}(O,C) = CNN(O,C) \in \{AD,NC\} \end{aligned}$$

where *CNN* represents the CNN (e.g., ResNet^31^, DenseNet^32^, EfficientNet^33^, Inception^34^), and $C \in {\mathbb {R}}^{2}$ denotes two clinical datapoints (i.e., age and sex), which are commonly used for AD diagnosis. We concatenate information *C* to the fully connected network input vector obtained from global average pooling. The CNN output is a two-dimensional probability vector, and the CNN is trained using the cross-entropy loss to determine the diagnostic probabilities for AD and NC.

#### Baseline 2: ML classifier using single radiomic feature

Baseline 2^35^ uses the FAZ area extracted from OCTA scans to diagnose AD. This is a radiomic method^36^ that uses only the FAZ area, which is only one of the available radiomic features. Consequently, baseline 2 uses a single radiomic feature, as illustrated in Fig. 2b. We train an ML model for binary classification of AD and NC, with the output using a 5D vector integrating the FAZ area and two clinical datapoints into the input vector for the ML model.

When using baseline 2, ophthalmologists should perform manual segmentation of the FAZ region on OCTA scan *O* to obtain FAZ binary mask $S^{ma}_O$ $\in {\{0,1\}^{h \times w}}$ (i.e., 0 and 1 for the outer and inner FAZ, respectively). The FAZ area is obtained by multiplying the number of nonzero pixels in $S_O$ (i.e., pixels located insider the FAZ) by constant *c* for the area per pixel as follows:

2 $$\begin{aligned} Area(S^{ma}_O) = \sum _{ij} c \times S^{ma}_O[i,j] \end{aligned}$$

Then, baseline 2 is trained to classify AD and NC through an ML algorithm using the FAZ area and two clinical datapoints as follows:

3 $$\begin{aligned} Baseline_{2}(O,C) = ML(Area(S^{ma}_O),C) \in \{AD,NC\} \end{aligned}$$

where ML$(\cdot )$ represents an ML model (e.g., XGBoost^37^, random forest^38^, LGBM^39^).

#### Proposed technique (AI-based segmentation with ML classifier using multiple radiomic features)

In contrast to baseline 2, as shown in Fig. 2c, the proposed technique simultaneously uses five representative radiomic features^40^ (i.e., area, roundness, eccentricity, compactness, and solidity) rather than simply considering the FAZ area. We experimentally demonstrate that multiple radiomic features increase the classifier diversity, thereby improving the AD diagnostic performance. In addition, unlike baseline 1, the proposed technique does not use an AI-based classifier but AI-based FAZ area segmentation. To obtain the binary mask for the FAZ area from an OCTA scan, AI-based segmentation is applied rather than the manual segmentation required for baseline 2. Hence, the proposed technique performs automatic AD diagnosis without pretreatment of OCTA scans, like in baseline 1. Moreover, AI-based FAZ segmentation mitigates annotation errors that may occur during manual FAZ segmentation, thereby increasing the accuracy of extracted radiomic features. Table 3 lists the characteristics of the evaluated techniques. Our proposal has a hybrid structure by combining AI and ML through the sequential execution of AI-based FAZ area segmentation and ML-based AD diagnosis based on multiple radiomic features extracted from the FAZ.

**Table 3:** Factor-specific differences between proposed and existing techniques.

Method	Segmentation	Feature	Classification	Comp model
Baseline 1	None	Automatically extracted	AI	AI
Baseline 2	Manual	Only area	ML	ML
Proposed	AI	Area + other features	ML	AI + ML

**Figure 3:**
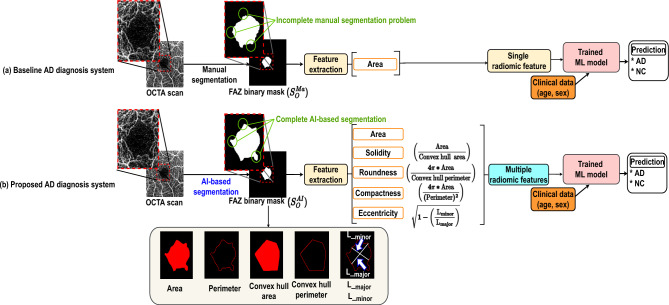
Feature extraction from FAZ segmented on OCTA scan. Multiple radiomic features are used for training in the proposed technique.

*Extraction of additional multiple radiomic features.* For comparison with baselines 1 and 2, four radiomic features were added to our technique, as defined below and illustrated in Fig. 3. Let $S \in \{0,1\}^{h \times w}$ be the FAZ binary segmentation mask.

- Solidity. The solidity measures the degree of curvature of the FAZ interface as the ratio of the FAZ inner area to its convex hull region:
  4 $$\begin{aligned} Solidity(S) = \frac{Area(S)}{Area_{cvh}(S)} \end{aligned}$$
  where *Area*(*S*) is the FAZ area (area in which *S* is 1) and $Area_{cvh}(S)$ is the FAZ convex hull area.
- Compactness. The compactness measures the degree of curvature of the FAZ interface as the ratio of the FAZ inner area to its perimeter:
  5 $$\begin{aligned} Compact(S) = \frac{4 \pi \cdot Area(S)}{p(S)^2} \end{aligned}$$
  where *p*(*S*) is the FAZ perimeter.
- Roundness. The roundness is similar to the compactness but uses the perimeter of the convex hull rather than the perimeter of the FAZ:
  6 $$\begin{aligned} Round(S) = \frac{4 \pi \cdot Area(S)}{p_{cvh}(S)^2} \end{aligned}$$
  where $p_{cvh}(S)$ is the perimeter of the FAZ convex hull.
- Eccentricity. The eccentricity is obtained as the ratio of the longest (*a*(*S*)) to the shortest (*b*(*S*)) straight-line length within the FAZ, *S*. It allows to measure the FAZ closeness to an ellipse as follows:
  7 $$\begin{aligned} Eccent(S) = \sqrt{{1 - \frac{b(S)^2}{a(S)^2}}}\ \,\, {(a(S) \ge b(S))} \end{aligned}$$

*AI-based FAZ segmentation.* Baseline 2 requires ophthalmologists to perform manual segmentation of the FAZ on OCTA scan *O* to obtain FAZ binary mask $S^{ma}_O$ $\in {\{0,1\}^{h \times w}}$. In contrast to baseline 2 with manual FAZ segmentation, the proposed technique uses an AI model to automatically segment the FAZ. Thus, the input is OCTA scan *O*, and the output is the extracted FAZ. For training, we used a public dataset, whereas our hospital data were used for evaluating FAZ segmentation and comparison with manual annotations in baseline 2. We denote the automatically segmented binary mask as $S^{AI}_O$ $\in {\{0,1\}^{h \times w}}$, with 0 and 1 indicating the outer and inner parts of the FAZ, respectively.

*Inference.* Using multiple radiomic features and automatic FAZ segmentation, the proposed technique performs inference as shown in Eq. (8), which is different from inference for the baselines given by Eqs. (1) and (3). In addition, the proposed technique learns to classify AD and NC through an ML model using the FAZ area, like in baseline 2, in addition to other four FAZ features.

8 $$\begin{aligned} Proposed(O,C) = ML(Area(S^{AI}_O),Solidity(S^{AI}_O),Compact(S^{AI}_O),Round(S^{AI}_O),Eccent(S^{AI}_O),C) \in \{AD,NC\} \end{aligned}$$

where ML$(\cdot )$ represents the same ML model used in baseline 2, $S^{AI}_O$ is the FAZ binary mask automatically extracted from OCTA scan *O*, and *C* represents the two clinical datapoints commonly used for all the techniques evaluated in this study.

## Evaluation metrics

To evaluate the proposed FAZ multiple radiomic features of Alzheimer’s diagnosis (binary classification of NC and AD), we used the area under the curve (AUC), accuracy, sensitivity, and specificity of the receiver operating characteristic (ROC) curve. For the ROC curve, we chose the most commonly used decision threshold of 0.5 and calculated the true positive (TP), true negative (TN), false positive (FP), and false negative (FN) rates based on this threshold. We then calculated the accuracy, sensitivity, and specificity values as follows:

9 $$\begin{aligned} Accuracy&= \frac{(TN + TP)}{(TN + TP + FN + FP)} ,\end{aligned}$$

10 $$\begin{aligned} Sensitivity&= \frac{(TP)}{(TP + FN)} ,\end{aligned}$$

11 $$\begin{aligned} Specificity&= \frac{(TN)}{(TN + FP)} , \end{aligned}$$

## Results

### Fivefold cross-validation results on the training set

We compared the diagnostic performance of the proposed technique and the baseline technique for AD diagnosis on the training set. We divided the training set into 85 OCTA scans, which were divided into five sets to apply fivefold cross-validation. Each diagnosis technique was trained five times, and the mean validation performance was considered as the final diagnostic performance. The training details for each technique are detailed below.

### Training details

#### Training for baseline 1

As the CNN backbone used in baseline 1, we tested four representative models: ResNet^31^, DenseNet^32^, EfficientNet^33^, and Inception^34^. Each model was trained with fivefold cross-validation using the pretraining parameters on the ImageNet dataset for initialization. Each training procedure proceeded for 50 epochs by applying the cross-entropy loss^41^ to a two-dimensional output probability vector for binary classification of positive (AD) and negative (NC) samples. The optimal learning rates were $1e^{-2}$ for EfficientNet^33^, $1e^{-2}$ for ResNet^31^, $1e^{-5}$ for Inception^34^, and $1e^{-2}$ for DenseNet^32^.

#### Training for baseline 2

Baseline 2 required manual extraction of FAZ binary mask $S^{ma}_O$ from an OCTA scan. Thus, ophthalmologists extracted the FAZ binary masks from the 85 OCTA scans used in this study. In baseline 2, given the FAZ binary mask $S^{ma}_O$ provided by the ophthalmologists, the area was calculated, and the ML model was applied to learn and evaluate the AD diagnosis by fivefold cross-validation. We used the LGBM^39^ as the ML model.

#### Training for proposed technique

**Figure 4:**
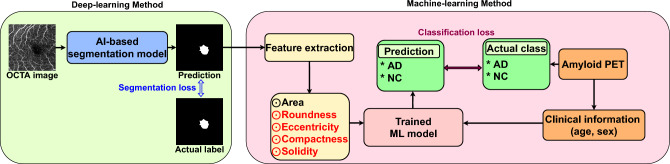
Training overview of proposed diagnosis technique. The proposed technique comprises AI-based FAZ segmentation and ML-based AD diagnosis using multiple radiomic FAZ features. Segmentation and classification loss functions are used as training losses for the AI and ML models, respectively. Data from our hospital are used for training and evaluation with fivefold cross-validation.

Unlike baseline 2, the proposed technique performs AI-based segmentation. It receives an OCTA scan as input and predicts the FAZ binary mask as the output. To train the segmentation model, we used 2000 OCTA scan–FAZ mask pairs^42^ based on nnUNet^43^. As the learning objective function, the conventional pixel-based cross-entropy loss was used for training over 100 epochs under Adam optimization^44^ with a learning rate of 0.01. Thus, 85 FAZ mask prediction results for the 85 evaluation OCTA scans were obtained from the learned segmentation model, and multiple radiomic features were extracted. Then, the ML model (LGBM^39^ for a fair comparison with baseline 2) was applied for AD diagnosis in fivefold cross-validation. Training of the proposed technique is illustrated in Fig. 4.

### Diagnostic performance

The diagnostic performance results (AUC) per fold and across folds of the evaluated techniques are listed in Table 4. The AUC of the proposed technique was at least 13% higher than that of the baselines. The baselines did not provide clinically meaningful results because all the AUC values were below 60%. In contrast, the proposed technique could achieve clinical significance with AUC values above 70%. Furthermore, the proposed method also demonstrates statistical significance with very low p-values when compared to the baselines, providing evidence of its statistical significance ($p < 0.05$). Hence, this is the first technique demonstrating that multiple radiomic FAZ features are meaningful biomarkers for AD diagnosis.

**Figure 5:**
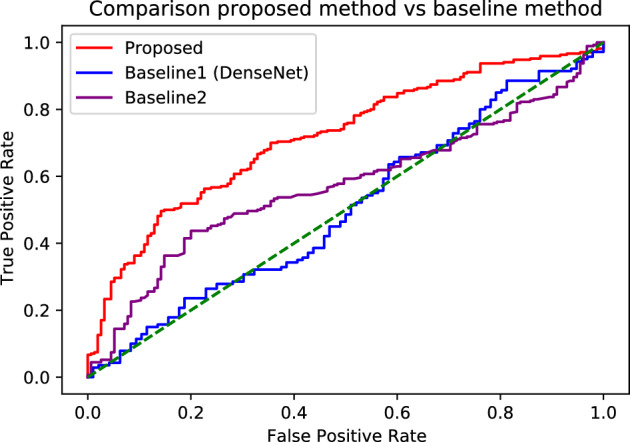
Receiver operating characteristic curves of differential diagnosis between AD and NC. The AUC values are $72.2\pm 4.2$ ($95\%$ confidence interval, $66.7-75.5$), $52.4\pm 3.9$ ($95\%$ confidence interval, $49-55.8$), and $59.1\pm 6.2$ ($95\%$ confidence interval, $52.9-65.3$) for the proposed technique, baseline 1 with DenseNet, and baseline 2, respectively.

Figure 5 shows the receiver operating characteristic curves of each technique for the aggregate AUC values derived from the cross-fold mean. In all the areas, regardless of the threshold, the proposed technique demonstrated higher sensitivity than the baselines, confirming its superiority.

**Table 4:** AUC of differential diagnosis between AD and NC. The mean and standard deviation are calculated from fivefold cross-validation.

Technique	Fold 1	Fold 2	Fold 3	Fold 4	Fold 5	Mean (%)	*p*-value
Baseline 1 (ResNet)^31^	50	47	58	45	55	$50.6\pm 4.4$	0.0001
Baseline 1 (DenseNet)^32^	51	49	60	52	50	$52.4\pm 3.9$	0.0001
Baseline 1 (EfficientNet)^33^	36	51	37	57	59	$48\pm 9.7$	0.0020
Baseline 1 (Inception)^34^	51	50	38	56	61	$51.2\pm 7.6$	0.0014
Baseline 2	51	64	49	60	68	$59.1\pm 7.1$	0.0126
**Proposed**	**76**	**76**	**65**	**70**	**72**	$\bf{{72.2}\pm {4.2}}$	−

### Analysis of proposed technique and its elements

#### Performance of different ML models

The proposed technique diagnosed AD by feeding multiple radiomic FAZ features into an ML classifier. The results using LGBM^39^ as a representative ML model are listed in Table 4, and the performance comparison of other ML models (i.e., XGBoost^37^ and random forest^38^) is presented in Table 5. LGBM showed the highest performance, thus being selected as the ML model for the proposed technique. For models other than LGBM, the mean diagnostic performance was at least 60%. Thus, the proposed technique showed higher performance than the baselines (AUC of 60% or less), as shown in Table 4. This demonstrates that the proposed method is superior to the baselines regardless of the underlying ML model.

**Table 5:** Performance of differential diagnosis between AD and NC using different ML models in the proposed technique. The mean and standard deviation are obtained from fivefold cross-validation.

Model	Fold 1	Fold 2	Fold 3	Fold 4	Fold 5	Mean (%)	*p*-value
XGBoost^37^	65	72	62	68	59	$65.5\pm 6.0$	0.0346
Random forest^38^	64	72	53	64	56	$62.3\pm 4.0$	0.0632
**LGBM**^39^	**76**	**76**	**65**	**70**	**72**	$\bf{{72.2}\pm {4.2}}$	−

#### Ablation study for proposed technique

The proposed technique uses multiple radiomic FAZ features (i.e., area, compactness, eccentricity, roundness, and solidity) instead of only one feature (i.e., area used in baseline 2). Table 6 shows that the diagnostic performance gradually improved when each of these features was added to the proposed technique. Hence, the diagnostic performance was improved by adding the four features to the area, justifying their inclusion in the technique.

**Table 6:** Comparison of AD diagnostic performance by including radiomic features. A, compactness; B, eccentricity; C, roundness; D, solidity. The mean and standard deviation are obtained from fivefold cross-validation.

Features	Fold 1	Fold 2	Fold 3	Fold 4	Fold 5	Mean (%)
*Area	54	69	62	60	69	$63.2\pm 5.0$
Area + A	57	71	60	61	69	$64.0\pm 5.4$ (+ 0.8)
Area + A + B	71	72	63	64	64	$67.2\pm 3.7$ (+ 4.0)
Area + A + B + C	67	74	63	69	68	$68.8\pm 3.3$ (+ 5.6)
**Area + A + B + C + D	**76**	**76**	**65**	**70**	**72**	$\bf{{72.2}\pm {4.2}\ (+ 9.0)}$

*Baseline 2 method (i.e., Using only area feature).

**Proposed method (i.e., Using five multiple radiomic features).

#### Validity of AI-based segmentation in proposed technique

The proposed approach automatically extracts the FAZ by AI-based segmentation. Table 7 lists the diagnostic performance when the proposed technique uses the binary masks manually annotated by an ophthalmologist, as in baseline 2, instead of the automatically segmented FAZ. The AUC of AD diagnosis was enhanced by 10.3% when AI-based automatic segmentation was used compared with manual segmentation. This performance improvement followed from the more accurate and precise AI-based FAZ extraction compared with manual annotations. We explain this performance improvement in section Discussion.

**Table 7:** AD diagnostic performance for manual and automatic segmentation. The mean and standard deviation are obtained from fivefold cross-validation.

Segmentation	Fold 1	Fold 2	Fold 3	Fold 4	Fold 5	Mean (%)
Manual	65	67	55	68	52	$61.9\pm 6.5$
**Automatic**	**76**	**76**	**65**	**70**	**72**	**72.2±4.2**$^*$ **(+ 10.3)**

*$$p<0.05$$.

### Comparison of diagnostic performance of proposed methods on holdout test set

We conducted a holdout test using a set of 45 OCTA scan holdout datasets. The holdout test was compared and verified by baseline^35^(i.e., baseline 2 method only area feature) and ophthalmologists, respectively. accuracy, sensitivity, specificity, and AUC measured the evaluation of each diagnostic technique.

#### Comparison diagnostic performance between baseline and proposed method

The diagnostic performance results for the baseline^35^ method (i.e., the baseline 2 method uses only a single area feature) and the proposed method (multiple radiomic features) are detailed in Table 8.

**Table 8:** Diagnostic performance comparison between proposed and baseline method. Accuracy, sensitivity, specificity, and AUC score obtained from the holdout test set. Values in parentheses represent improvements in performance between the proposed and baseline method diagnoses. The mean and standard deviation are obtained from the holdout test.

Method	Accuracy (%)	Sensitivity (%)	Specificity (%)	AUC (%)
Baseline	50.7±5.5	35.2±15.6	78.7±13.5	58.0±0.9
**Proposed**	**64.8±4.3**$^*$ **(+ 14.1)**	**54.4±5.0**$^*$ **(+ 19.2)**	**83.7±6.3**$^*$ **(+ 5.0)**	**72.0±4.8**$^*$ **(+ 14)**

* $$p<0.05$$.

FAZ binary masks were obtained manually and automatically (i.e., AI-based segmentation) for a holdout dataset for both the baseline and the proposed method. The FAZ binary masks were then used to test pre-trained models through a fivefold cross-validation process. In the holdout test, the proposed method showed a significant improvement in AUC compared to the baseline, with an improvement of 14% (baseline AUC 58.0% vs proposed AUC 72.0%). Furthermore, when compared to the baseline, the proposed method showed a 14.1% increase in accuracy (baseline accuracy 50.7% vs proposed accuracy 64.8%), a 5.0% increase in specificity (baseline specificity 78.7% vs proposed specificity 83.7%), and a 19.2% increase in sensitivity (baseline sensitivity 35.2% vs proposed sensitivity 54.4%). These results demonstrate the robustness of the proposed method in the holdout test, demonstrating superior performance in all evaluation metrics compared to the baseline ($p < 0.05$). Therefore, the proposed method demonstrates excellent diagnostic performance for the diagnosis of Alzheimer’s disease based on FAZ.

#### Comparison diagnostic performance between the ophthalmologist and proposed method

diagnosis of humans was conducted with the evaluation of three experienced ophthalmologists who did not participate in the collection of the OCTA holdout test dataset. For the diagnosis of humans, training was performed using the FAZ binary mask of 85 labeled training sets, and subsequently, an evaluation was performed using the FAZ binary mask of 45 unlabeled holdout datasets. The results of comparing the proposed method with the ophthalmologists are presented in Table 9.

**Table 9:** Diagnostic performance comparison between proposed method and ophthalmologist. Accuracy, sensitivity, specificity, and AUC score obtained from the holdout test set. Values in parentheses represent improvements in performance between the proposed method and ophthalmologist diagnoses. The mean and standard deviation are obtained from the holdout test.

Method	Accuracy (%)	Sensitivity (%)	Specificity (%)	AUC (%)
Humans	53.1±15	52.8±14.1	53.7±18.8	53.2±21.0
**Proposed**	**64.8±4.3**$^*$ **(+ 11.7)**	**54.4±5.0**$^*$ **(+ 1.6)**	**83.7±6.3**$^*$ **(+ 30.0)**	**72.0±4.8**$^*$ **(+ 18.8)**

* $$p<0.05$$.

The proposed method showed superior performance in all metrics (sensitivity, specificity, accuracy, and AUC) compared to ophthalmologists, particularly showing a significant improvement of over 30% in specificity ($p < 0.05$). This suggests that the proposed method is more sensitive in reducing false positives compared to ophthalmologists (humans’ specificity 53.7% vs proposed specificity 83.7%). In other words, it can significantly reduce the rate of false positive predictions for normal patients, which is cost-effective by saving on additional testing expenses (humans’ specificity 53.7% vs proposed specificity 83.7%). Furthermore, the proposed technique demonstrated higher sensitivity compared to ophthalmologists (humans’ sensitivity 52.8% vs proposed sensitivity 54.4%) and showed strong discriminative power for false negatives (humans’ AUC 53.2% vs proposed AUC 72.0%). Consequently, the proposed method shows potential utility as a clinical support tool for Alzheimer’s diagnoses based on FAZ in the future.

**Figure 6:**
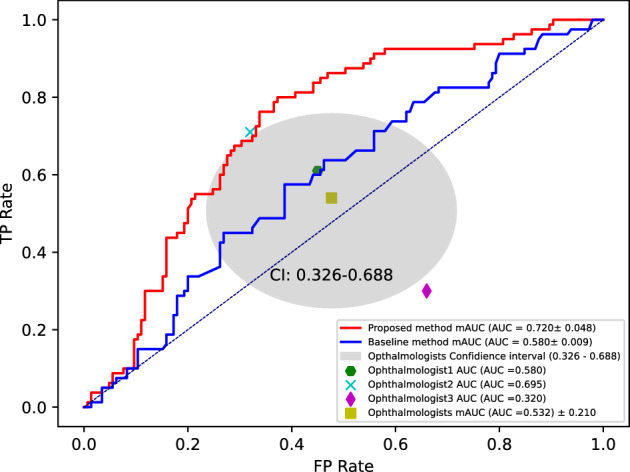
Comparison diagnostic performance of the proposed method and average of AUCs for three ophthalmologists on holdout test. The AUC values are $72.0\pm 4.8$ ($95\%$ confidence interval, $67.7-76.2$), $58.0\pm 0.009$ ($95\%$ confidence interval, $57.9-58.0$), and $53.2\pm 21.0\%$ ($95\%$ confidence interval, $32.0-69.5$) for the proposed technique, baseline, and average of AUCs for three ophthalmologists.

Figure 6 shows the AUC results for the binary classification of AD and NC using the proposed method and three ophthalmologists. The proposed method yields results that are 18.8% higher than the average AUC of the three ophthalmologists (humans’ AUC 53.2% vs proposed AUC 72.0%). In addition, the AUC of the proposed method showed a 14% improvement compared to the baseline (baseline AUC 58.0% vs proposed AUC 72.0%). This confirms that the proposed method (i.e., using multiple radiomics features including area) exhibited a significant performance improvement by considering multiple radiomics features, in contrast to the baseline method that relied on a single feature (i.e., using only area) for Alzheimer’s diagnosis (baseline AUC 58.0% vs proposed AUC 72.0%). Notably, a significant performance improvement was achieved even when compared to ophthalmologists (humans’ AUC 53.2% vs proposed AUC 72.0%). This indicates the potential of multiple radiomics features as a novel biomarker in FAZ-based Alzheimer’s diagnosis.

### Discussion

We showed that multiple radiomic FAZ features can be extracted by an AI model to support AD diagnosis. To the best of our knowledge, this is the first report using multiple radiomic FAZ features for diagnosis in patients with AD. We developed an automatic AD diagnosis technique comprising AI-based FAZ segmentation and ML-based AD diagnosis using the automatically extracted FAZ features.

#### Clinical implications of multiple radiomic FAZ features

Early detection of AD is of paramount importance, as it allows intervention prior to the onset of irreversible brain degeneration. Nevertheless, the current gold-standard diagnostic methods for AD, such as amyloid PET scans or CSF analysis, are insufficient as early screening tools. The retina, due to its embryological similarities with the brain and its easily and safely examined anatomical features, presents a promising avenue for the early detection of AD. The FAZ is a potential retinal biomarker for AD. The FAZ can be extracted from OCTA, which is a noninvasive retinal imaging modality. A recent meta-analysis revealed an enlargement of the FAZ in AD^14^. Another meta-analysis reported an enlarged FAZ in patients with mild cognitive impairment but no significant enlargement in AD^45^, while another meta-analysis showed no significant enlargement of the FAZ in AD^46^. Although limitations included the heterogeneity of OCTA equipment, diverse scanning protocols, and unmeasured confounders, previous studies only investigated the FAZ area, neglecting the FAZ shape, which may be a reliable indicator of retinal disorders^15,17,47^.

While area is frequently utilized as a primary metric for characterizing the FAZ, it is essential to recognize the substantial normal variation in FAZ size^48^. This variability may potentially constrain its utility as a pathological indicator in cross-sectional screening applications^49^. Evaluating the regularity of the FAZ’s overall shape, measured in terms of roundness or circularity, may offer a more precise indication of disease due to reduced variability within the healthy population^50^. Consequently, it is imperative to investigate whether biomarkers related to the shape of the FAZ possess diagnostic capabilities in individuals with AD. Recently, not only have there been reports of studies using ophthalmic imaging and AI for the diagnosis of ophthalmic diseases, but there have also been reports on their use for diagnosing AD^29,51–53^. In this study, we first revealed that multiple radiomic FAZ features, including roundness, eccentricity, compactness, and solidity, can improve the AD diagnostic performance compared with the FAZ area alone. Therefore, multiple radiomic FAZ features are useful for diagnosis and should be considered when evaluating the FAZ as new biomarkers for AD.

While our advanced AI-based methodology demonstrated a successful diagnosis of AD with a favorable diagnostic accuracy of “$72.2\pm 4.2\%$,” it is important to acknowledge that this figure falls short of direct comparison with current gold standard diagnostic methods. Notably, our diagnosis was solely based on retinal imaging, without the utilization of traditionally established diagnostic tools for AD, such as amyloid PET scans, CSF tapping, brain imaging, and even the Mini-Mental State Examination. Nevertheless, the findings from our study have significant clinical implications. They bridge a well-recognized diagnostic gap by providing a non-invasive and cost-effective means for screening AD, circumventing the need for invasive and expensive tests like PET, CSF tapping, and brain MRI. This innovative approach not only offers potential clinical utility but also signals a promising avenue for further refinement. Moreover, our study’s results indicate the potential for further refinement of AI-based diagnostic techniques, which holds promise for future research endeavors focused on enhancing the early detection of AD. This work not only contributes to the field’s knowledge but also paves the way for continued exploration and development in the realm of AD diagnosis.

#### Possible mechanisms for FAZ changes in AD

Vascular dysfunction in patients with AD likely leads to cerebral hypoperfusion during AD development^54–58^. In vivo and autopsy data have revealed that AD is associated with the deposition of amyloid and collagen within the cerebral capillaries, which can result in cellular apoptosis and vessel dropout^59–62^. In addition, various studies have found the accumulation of beta-amyloid plaques in the inner retina of postmortem tissue extracted from patients with AD^63–66^. Therefore, FAZ changes in patients with AD may be secondary to retinal degeneration owing to beta-amyloid accumulation within the retina.

#### Performance improvement by AI-based FAZ segmentation

**Figure 7:**
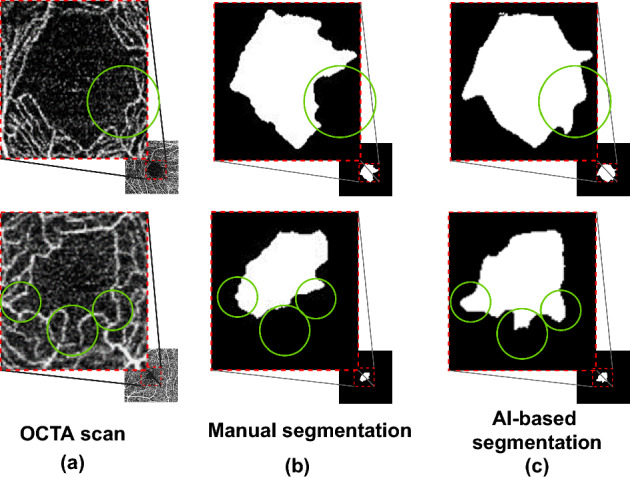
Comparison between FAZ segmentation methods. (**a**) Original OCTA scan and results from (**b**) manual and (**c**) AI-based segmentation.

To evaluate the effectiveness of AI-based FAZ segmentation integrated into the proposed technique, we compared it with manual FAZ segmentation, obtaining the results listed in Table 7. Manual segmentation was the same as that in baseline 2. Compared with manual segmentation, AI-based segmentation improved the diagnostic performance in terms of AUC from 61.9 to 72.2 (improvement of 10.3$\%$). As shown in Fig. 7, the performance improvement was due to AI-based FAZ segmentation overcoming problems and errors in manual annotation, which showed some inaccurate or mistaken results. Nguyen et al.^67^ reported the high performance of AI-based FAZ segmentation. We observed that the AI-based FAZ segmentation extracted the FAZ more precisely. Thus, the multiple radiomic FAZ features were more precisely determined, thereby improving AD diagnosis.

#### Performance improvement by multiple radiomic features

Different from previous studies^35^, we considered multiple FAZ features (i.e., area, roundness, eccentricity, compactness, and solidity) to diagnose AD. Existing techniques relied only on the FAZ area, and their AD diagnostic performance was not high. We demonstrated that various FAZ features contributed to further improving AD diagnosis, as indicated in Table 10. Every feature considered in this study (i.e., roundness, eccentricity, compactness, and solidity) contributed to the diagnosis, individually leading to comparable performance to that of the area. This individual validation may indicate the diagnostic performance improvement achieved by feature combination, as shown in Table 5, with the performance gradually improving as more features were added.

**Table 10:** AD diagnostic performance obtained from every radiomic feature. The mean and standard deviation are obtained from fivefold cross-validation.

Feature	Fold 1	Fold 2	Fold 3	Fold 4	Fold 5	Mean (%)
Area	54	69	62	60	69	$63.2\pm 5.0$
Solidity	59	75	50	66	71	$64.6\pm 8.0$
Roundness	54	68	47	62	66	$59.9\pm 7.0$
Compactness	60	67	58	72	71	$66.0\pm 5.0$
Eccentricity	63	78	61	53	60	$63.5\pm 8.0$
**All features**	76	76	65	70	72	$\bf{{72.2}\pm {4.2}}$

#### Technical implications

Our hybrid technique achieved an AUC of 72.2%, thus improving the AD diagnostic performance using FAZ biomarkers by 13.1% compared with existing techniques. This result holds notable clinical significance because it confirms that the FAZ is a suitable biomarker for AD, even though it was previously overlooked due to its low diagnostic performance in AD diagnosis. Hence, high AD diagnostic performance may be achieved by using FAZ biomarkers along with well-known biomarkers (e.g., global retinal nerve fiber layer, retinal thickness, vascular density, and FAZ area) that have been used for noninvasive AD diagnosis.

#### Promising performance of additional features in isolated feature analysis

We conducted comparative experiments by individually diagnosing isolated features, including the previously reported area^35^ feature and the four additional multiple features introduced in this study (i.e., solidity, compactness, eccentricity, roundness). The results for each of these isolated single features are detailed in Table 10.

Compared to the area, which was previously reported in FAZ-based Alzheimer’s diagnosis, the additional multiple features demonstrated their diagnostic potential, with solidity at 64.6% (+ 1.4%), roundness at 59.9% ($-$ 3.3%), compactness at 66.0% (+ 2.8%) and eccentricity at 63.5% (+ 0.3%) in the single feature comparison experiments. This confirms the excellent performance of the majority of these features. Furthermore, it suggests that these multiple features (i.e., solidity, compactness, eccentricity, roundness) have significant correlations with structural changes in FAZ caused by Alzheimer’s disease, beyond only area. This research not only contributes to the significant impact of FAZ-based Alzheimer’s diagnosis but also provides the first study presenting meaningful biomarkers for detecting structural changes due to other ocular diseases.

#### Instrumental applicability of the proposed method in real clinical settings

The proposed method showed a high-specificity model, but it was possible to derive a model with high sensitivity by adjusting the thresholds. Threshold adjustment resulted in a sensitivity of 90% and a specificity of 33%. This means that it can identify 90% of Alzheimer’s patients while detecting around 30% of the normal control group. Specifically, it excels at accurately detecting 90% of Alzheimer’s patients, enabling them to be referred for secondary testing such as amyloid PET scans and CSF analysis. At the same time, it provides a basis for reducing the cost of secondary testing in around 30% of normal patients. This is because, unlike ophthalmologists, the proposed technique uses AI technology to achieve a model with high sensitivity through various threshold adjustments. As a result, we can provide a model that, for the first time, detects up to 90% of actual Alzheimer’s patients while providing a false positive rate of less than 10%.

#### Comparison between proposed and existing techniques

We compared and analyzed the differences between the proposed and existing techniques regarding various aspects, as summarized in Table 11.

OCTA provides scans in a short time, enabling efficient noninvasive FAZ analysis. In only one other study, OCTA was used for AD diagnosis (third column of Table 11)^35^. However, that study used only the FAZ area, discarding other radiomic features. We demonstrated the importance of using multiple FAZ features for AD diagnosis by improving the diagnostic performance when using the proposed technique compared with conventional techniques that use a single feature (i.e., baseline 2).

Among existing studies using OCTA, Chan et al.^68^ and Mirshahi et al.^50^ used AI-based segmentation to extract the FAZ (fourth column of Table 11). They reported that using AI enabled the extraction of FAZ boundaries with better accuracy than existing signal processing methods, thereby validating the use of AI-based FAZ segmentation in our technique. However, the contribution of the extracted FAZ to diagnosis was not confirmed in those studies. Our study has both technical and clinical significance because we showed that AI-based FAZ segmentation can improve the diagnostic performance for AD.

Shiihara et al.^69^ and Philip et al.^70^ extracted multiple FAZ features like in our study (fifth column of Table 11). However, they did not develop an ML model for diagnosing a specific disease using multiple FAZ features (sixth column of Table 11). Shiihara et al.^69^ found a small difference between individuals in other FAZ features in addition to the area for healthy subjects, thereby suggesting their potential as biomarkers. However, their study was limited to healthy subjects, without confirming the possibility of using multiple biomarkers for diagnosing specific diseases. In contrast, we demonstrated that features other than the FAZ area are useful biomarkers for AD and developed an ML model for diagnosis. Philip et al.^70^ analyzed whether multiple FAZ features were individually correlated with primary open-angle glaucoma or exfoliation glaucoma, but they did not observe a correlation with a specific disease by combining multiple features. Therefore, they did not validate feature combinations. In addition, they did not implement a technique for disease diagnosis taking those features as inputs. Our study provides clinical and technical significance by overcoming existing limitations in AD diagnosis by implementing an ML model that receives multiple radiomic FAZ features as inputs and provides the AD diagnosis result as output.

**Table 11:** Characteristic of proposed and existing techniques. Unlike previous studies, our study covers all the listed aspects.

Study	OCTA scans as input	Target disease	AI-based segmentation?	Multiple FAZ features	Diagnosis considering multiple FAZ features
Werner et al.^71^	O	Retinal vein occlusion.	X	X	X
Shiihara et al.^69^	O	NC	X	O	X
Philip et al.^70^	O	Exfoliation glaucoma Primary open-angle glaucoma	X	O	X
Chan et al.^68^	O	Diabetic retinopathy	O	X	X
Mirshahi et al.^50^	O	Diabetic retinopathy	O	X	X
O’Bryhim et al.^35^	O	AD	X	X	X
**This study**	**O**	**AD**	**O**	**O**	**O**

#### Limitations

A major limitation of our study was the small sample size, which consisted solely of Asian individuals. Nevertheless, to overcome this limitation, a fivefold cross-validation and a holdout test were applied in the paper. The limitation of this holdout test is that it relies on data from a single institution and lacks external validation. However, in our study, we tried to collect data for the fivefold cross-validation experiment and the holdout test at different periods, attempting to separate the data as effectively as possible. Furthermore, the exclusion of patients with known vascular disease from our study was another limitation. We could not evaluate whether these results are applicable to individuals who may have retinal microvascular alterations by other causes. In addition, the inclusion of participants with cognitive changes and positive biomarkers for AD limited comparisons with subjects with preclinical and positive biomarkers, such as mild cognitive impairment. Nevertheless, we demonstrated the diagnostic ability of FAZ with AI for AD and all individuals in this study, including those in the AD and NC groups, were screened by amyloid PET. In future work, a comparison between patients with mild cognitive impairment and NCs and longitudinal changes in FAZ in AD will be considered.

## Conclusion

Employing an advanced AI-based methodology, we successfully automated FAZ segmentation and extracted a comprehensive array of radiomic FAZ features. The integration of FAZ area with these additional features presents a promising avenue for the development of robust and potentially transformative biomarkers for AD. Furthermore, our AI-driven FAZ analysis, encompassing automatic segmentation and multi-feature extraction, not only holds substantial promise for AD diagnosis but also extends its utility to the broader spectrum of retinal disorders, underlining its pivotal role in advancing clinical ophthalmology and neurology.

## Data Availability

The main data supporting the results of this study are available within the paper. The raw datasets from Samsung Medical Center are protected to preserve patient privacy, but they can be made available upon reasonable request if approval is obtained from the corresponding Institutional Review Board. For the request, please contact Don-Il Ham (di.ham@samsung.com).

## Code availability

The code used in this study is available at https://github.com/kskim-phd/Hybrid-FAZ, which will be disclosed upon the publication or second/revised submission.
